# Diabetes and COVID-19 Outcomes: An Analysis of Freeman Health System Patients

**DOI:** 10.7759/cureus.54249

**Published:** 2024-02-15

**Authors:** Timothy Wiant, Logan Schmidt, SaiBhavana Srikakolapu, Nova Beyersdorfer, Mariam Akhtar, Kerry Johnson, Greg Stahl, Darrin S Goade, Robert D Arnce

**Affiliations:** 1 College of Medicine, Kansas City University, Kansas City, USA; 2 College of Medicine, Kansas City University, Joplin, USA; 3 Mathematics, Missouri Southern State University, Joplin, USA; 4 Pharmacy, Freeman Health System, Joplin, USA

**Keywords:** type 1 and type 2 diabetes mellitus, mortality, rural population, diabetes, covid-19

## Abstract

Background: As COVID-19 continues to affect millions of people around the world, it has become vital to understand how comorbidities such as diabetes affect the health outcomes of these patients. While earlier studies have focused on major metropolitan areas, rural settings have been comparatively understudied. The goal of this study is to understand the effect on mortality that these two diseases have in the inpatient setting of a rural population.

Methods: The electronic medical records of all adult patients admitted to Freeman Health System, Joplin, Missouri, United States, between April 1, 2020, and December 31, 2021, were reviewed for the presence of COVID-19 infection and/or diabetes (type I and type II). Freeman Health is a major health system headquartered in Southwest Missouri. Diagnoses were obtained through the use of standard International Classification of Disease, 10^th^ edition (ICD-10) codes. The initial data set consisted of 19,323 admissions. After excluding duplicate admissions and those who had already been infected with COVID-19, 1,729 patients with COVID-19, 172 patients with type I diabetes, and 3,992 patients with type II diabetes were included in the analysis of inpatient all-cause mortality. We hypothesized that patients with type I and type II diabetes would both show an increased risk of all-cause mortality. Mortality in the context of our study results refers to all-cause mortality.

Results: The all-cause mortality rate was 19.94% (137/687, with a 95% confidence interval (CI) of 16.95%-22.93%) in patients admitted with both diabetes (the combined type I and type II subsets) and COVID-19 (P1). The mortality rate was 16.03% (167/1042, with 95% CI of 13.80%-18.25%) in patients admitted with COVID-19 who did not have diabetes (P2). Patients admitted with a comorbid diagnosis of diabetes but without COVID-19 (P5) had a much lower mortality rate of 5.98% (249/4164, with a 95% CI of 5.26%-6.70%). The combination of both COVID-19 and diabetes together was associated with a higher mortality rate than either of the two separately. The mortality rate was additionally elevated in patients with both type II diabetes and COVID-19 (P4) (134/663, mortality rate of 20.21% with 95% CI of 17.15%-23.27%) versus those with COVID-19 without diabetes (P2) (167/1042, 16.03% with 95% CI of 13.80%-18.25%), an overall difference of 4.18% (95% CI of 0.40%-7.94%). The subset of patients with type I diabetes with COVID-19 (P3) and type I diabetes without COVID-19 (P6) were too small to accurately power individual analysis. The subset of patients with diabetes (type I and type II) and without COVID-19 (P5) had the lowest mortality rate of any subset adequately powered for analysis at 5.98% (249/41464, CI of 5.26%-6.70%).

Conclusions: The results of this study show that type II diabetes is a significant risk factor for mortality in admitted COVID-19 patients. P4 had the highest overall mortality of any subset studied. The study was underpowered to show if type I diabetes patients, with and without COVID-19, had an increased mortality when analyzed separately. COVID-19 significantly increased mortality in all subsets adequately powered for full analysis.

## Introduction

While the worst days of the COVID-19 pandemic may be behind us, diabetes continues to increase in prevalence worldwide [1]. An analysis conducted by the CDC indicated that patients with diabetes accounted for 39.5% of all COVID-19 deaths in the United States and 49.6% of COVID-19 deaths in patients under the age of 65 [2]. The overall death toll continues to rise; the United States saw more than 1.15 million COVID-19 deaths by October 2023 [3]. In 2021, the International Diabetes Foundation (IDF) reported that diabetes affects 10.5% of 20-79-year-olds in the world, putting the total number of affected at 536.6 million people. In the same article, the IDF predicts this number will rise to 783.2 million individuals by 2045 [1]. Diabetes has been used as a risk factor in patients diagnosed with COVID-19 to delegate the distribution of resources, as well as to develop the standard of care for treatment. Many previous studies of the relationship between COVID-19 and diabetes came from patient populations other than from the rural areas of the United States [4,5]. During the initial COVID-19 vaccine rollout, diabetes diagnosis helped to determine priority for receiving COVID-19 vaccines [6]. Diabetes was used as a risk factor to determine monoclonal antibody eligibility in the past [7], and continues to be used by CDC guidelines for determining eligibility for COVID-19 antivirals such as nirmatrelvir/ritonavir (Paxlovid) [8].

As the scientific community navigates the long-term consequences of COVID-19, we conducted this study seeking to better understand the relationship between diabetes and COVID-19 mortality. Previous meta-analyses have suggested that diabetes is the single most important cause of mortality in hospitalized COVID-19 patients, finding a 1.85 times increase in the risk of death for COVID-19 patients with diabetes [9]. Current research in mice has shown that diabetic patients have increased expression of the angiotensin-converting enzyme 2 (ACE2) receptor. The ACE2 receptor is most commonly associated with alveolar type I and type II epithelial cells but has also been found in multiple organs including the pancreas. This receptor was found to be a target in COVID-19-related viral infections [10]. This relationship may provide an understanding of why diabetic patients are more predisposed to severe COVID-19 infection. The exact relationship between diabetes and COVID-19 mortality has varied based on the study type and the population [4,5]. To date, the relationship between COVID-19 mortality and diabetes has been understudied in the rural areas of the United States. The previous studies have generally focused on larger metro areas or overseas populations, making our study important in helping to bridge this gap of data [5,11,12]. 

An abbreviated version of this study was previously presented as an abstract at the 2023 Kansas City University (KCU) Research Symposium in Joplin, Missouri, United States, on April 6, 2023. 

## Materials and methods

Objectives 

The purpose of this study is to compare mortality outcomes of COVID-19 patients with and without type I and type II diabetes in a rural population. A prior study conducted in the early days of the COVID-19 pandemic indicates that COVID-19 patients with diabetes/ uncontrolled hyperglycemia had a markedly higher mortality rate than COVID-19 patients without diabetes/ uncontrolled hyperglycemia (28.8% vs. 6.2%, respectively, p < 0.001) [13]. In a previous study looking into the effects of various social determinants of health on COVID-19 mortality, it was found that the mortality rate was 65.43 per 100,000 in urban counties, and 50.78 per 100,000 in rural counties (p-value < 0.0001) [14]. However, this study was conducted in the earlier stages of the pandemic, and rural patients face challenges such as lower median income and increased distance to healthcare facilities [15]. Additionally, the rate of vaccinated individuals is much lower in rural vs. urban communities (59% vs. 75%, respectively) and this gap has continued to grow larger since vaccines were made widely available to all adults in April 2021 [16]. In England, one study found that those with type I diabetes and COVID-19 had a higher odds ratio of in-hospital mortality than those with type II diabetes with COVID-19 [17].

Our study expanded on this finding to determine whether the comorbid subsets have a higher mortality rate than the subsets without a diagnosis of diabetes, and if the combination of COVID-19 and diabetes (P1) has a higher mortality than the individual populations of COVID-19 (P2) and diabetes (P5), respectively. Additionally, our study sought to determine if the subset of diabetes, type I or type II, has an impact on overall mortality. We hypothesized that patients with diabetes will suffer a higher mortality rate when infected with COVID-19, compared to patients who have diabetes but not COVID-19 and compared to patients with COVID-19 but not diabetes.

Methods 

A retrospective analysis was conducted at Freeman Health for persons admitted between April 1, 2020, and December 31, 2021. Data was collected via electronic medical records. Diagnoses were obtained through the use of standard International Classification of Disease, 10th edition (ICD-10) codes. Freeman Health has 410 licensed beds in Joplin, Missouri, United States, and 25 in Neosho, Missouri, United States. Joplin is the largest city in the Joplin Metropolitan Area with a population of over 50,000 [11]. Research approval was obtained through the Freeman Health System Institutional Review Board with Approval Number 202201.

ICD-10 designations showed that of the study population analyzed, 1.2% had type I diabetes, 28.1% had type II diabetes, and 10.4% had COVID-19 once duplicates and prior admissions were removed. All patient identifiers were removed, but age, date of admittance and discharge, race, sex, and insurance payers were recorded; these were not used as inclusion or exclusion criteria for the study. Due to the retrospective nature of the study, informed consent was not required. The major categorical variable is the dependent variable of all-cause mortality. All-cause mortality was measured by the number of patients that expired within their subset and subsequently reported as a mortality rate with a 95% confidence interval (CI). The average age of COVID-19 patients was 49.04 years for those with type I diabetes, 66.37 years for type II diabetes, and 60.93 years for patients without diabetes. Due to the retrospective study design, the samples were not chosen randomly from their respective populations and therefore may not be completely representative of their respective populations. The study took place from April 1, 2020, to December 31, 2021. The ICD-10 codes used and the subsets analyzed in the study can be seen in Table 1 and Table 2 below. Table 3 shows the abbreviation used for each subset. This study included patients admitted with a comorbid COVID-19 or diabetes diagnosis even if their primary admitting diagnosis represented another condition.

**Table 1:** COVID-19 ICD-10 diagnosis code used ICD: International Classification of Diseases

ICD-10 code	Diagnosis
U071	COVID-19

**Table 2:** ICD-10 diabetes diagnoses ICD: International Classification of Diseases E10* and E11* represent that all subgroups that start with E10/E11 were included, i.e., in this case, all type I and type II diabetes diagnoses.

ICD-10 code	Diagnosis
E10*	Type 1 diabetes mellitus
E11*	Type 2 diabetes mellitus
E10.10	Type 1 diabetes mellitus with ketoacidosis without coma
E10.22	Type 1 diabetes mellitus with diabetic chronic kidney disease
E10.319	Type 1 diabetes mellitus with unspecified diabetic retinopathy without macular edema
E10.40	Type 1 diabetes mellitus with diabetic neuropathy, unspecified
E10.42	Type 1 diabetes mellitus with diabetic polyneuropathy
E10.43	Type 1 diabetes mellitus with diabetic autonomic (poly)neuropathy
E10.51	Type 1 diabetes mellitus with diabetic peripheral angiopathy without gangrene
E10.649	Type 1 diabetes mellitus with hypoglycemia without coma
E10.65	Type 1 diabetes mellitus with hyperglycemia
E10.9	Type 1 diabetes mellitus without complications
E11.10	Type 2 diabetes mellitus with ketoacidosis without coma
E11.21	Type 2 diabetes mellitus with diabetic nephropathy
E11.22	Type 2 diabetes mellitus with diabetic chronic kidney disease
E11.319	Type 2 diabetes mellitus with unspecified diabetic retinopathy without macular edema
E11.36	Type 2 diabetes mellitus with diabetic cataract
E11.39	Type 2 diabetes mellitus with other diabetic ophthalmic complication
E11.40	Type 2 diabetes mellitus with diabetic neuropathy, unspecified
E11.42	Type 2 diabetes mellitus with diabetic polyneuropathy
E11.43	Type 2 diabetes mellitus with diabetic autonomic (poly)neuropathy
E11.51	Type 2 diabetes mellitus with diabetic peripheral angiopathy without gangrene
E11.52	Type 2 diabetes mellitus with diabetic peripheral angiopathy with gangrene
E11.621	Type 2 diabetes mellitus with foot ulcer
E11.622	Type 2 diabetes mellitus with other skin ulcer
E11.641	Type 2 diabetes mellitus with hypoglycemia with coma
E11.649	Type 2 diabetes mellitus with hypoglycemia without coma
E11.65	Type 2 diabetes mellitus with hyperglycemia
E11.69	Type 2 diabetes mellitus with other specified complication
E11.9	Type 2 diabetes mellitus without complications
E13.9	Other specified diabetes mellitus without complications

**Table 3:** Population subsets

	Population	Number of patients
P1	Patients with both COVID-19 and diabetes	687
P2	Patients with COVID-19 and without diabetes	1042
P3	Patients with COVID-19 and type I diabetes	24
P4	Patients with COVID-19 and type II diabetes	663
P5	Patients with diabetes and without COVID-19	4164
P6	Patients with type I diabetes and without COVID-19	172
P7	Patients with type II diabetes and without COVID-19	3992

Statistical methods 

Our total sample consisted of 19,323 admissions, of whom 17,540 did not have COVID-19 and 1,783 were COVID-19 positive (ICD-10 code U071). After removing duplicate admissions and patients who had already presented with COVID-19, the total number of patients included in the study was 16,579, with 14,850 non-COVID-19 patients and 1,729 COVID-19 patients (representing 94.71% and 10.43% of the study total, respectively). The patients who were previously diagnosed with COVID-19 were excluded so that the effects of prior illness and subsequent development of immunity did not affect the results. There were 4,164 patients included in P5. Of the 4,164 patients, 172/4164 (4.13%) had type I diabetes, and 3,992/4164 (95.87%) had type II diabetes; these patients will henceforth be referred to as the subsets P6 and P7, respectively.

After removing duplicates, the 1729 patients presenting with COVID-19 were analyzed similarly. A total of 687/1729 patients were included in P1 and 1,042/1729 patients were included in P2. Of the 687 patients included in P1, 24/687 (3.49%) had type I diabetes (P3), and 663/687 (96.51%) had type II diabetes (P4). Our subsets were analyzed using two-sample proportion summary hypothesis tests to compare all-cause mortality between our subsets.

## Results

The mortality rate of P1 was 19.94% (95% CI: 16.95%-22.93%, 137/687), of P2 was 16.03% (95% CI: 13.80%-18.25%, 167/1042), of P4 was 20.21% (95% CI: 17.15%-23.27%, 134/663), of P5 was 5.98% (95% CI: 5.26%-6.70%, 249/4164), and of P7 was 6.06% (95% CI: 5.32%-6.80%, 242/3992) (Table 4). Subsets P3 and P6 were not able to yield significant data due to the small size of the sample, and thus mortality rates were not factored into subsequent individual analyses. The mortality rates ranged from a low of 5.98% (249 of 4164) for P5 to 20.21% (134 of 663) for P4.

**Table 4:** Confidence intervals (CIs) for individual population proportions P1: Patients with both COVID-19 and diabetes; P2: Patients with COVID-19 and without diabetes; P3: Patients with COVID-19 and type I diabetes; P4: Patients with COVID-19 and type II diabetes; P5: Patients with diabetes and without COVID-19; P6: Patients with type I diabetes and without COVID-19; P7: Patients with type II diabetes and without COVID-19 Mortality (N) represents the number of persons in each subset who expired. It is represented here out of the number of persons in each subset (the sample proportion), e.g., in P1, 137 out of a total of 687 persons expired. Subset mortality represents the proportion of each subset that expired in a percentage format. The 95% CIs of the sample proportions are represented in the final column.

Subset mortality (N)	Subset mortality percentage	95% CI
P1- 137 of 687	19.94%	16.95% - 22.93%
P2- 167 of 1042	16.03%	13.80% - 18.25%
P3- 3 of 24	12.50%	N/A
P4- 134 of 663	20.21%	17.15% - 23.27%
P5- 249 of 4164	5.98%	5.26% - 6.70%
P6- 7 of 172	4.07%	N/A
P7- 242 of 3992	6.06%	5.32% - 6.80%

**Figure 1:**
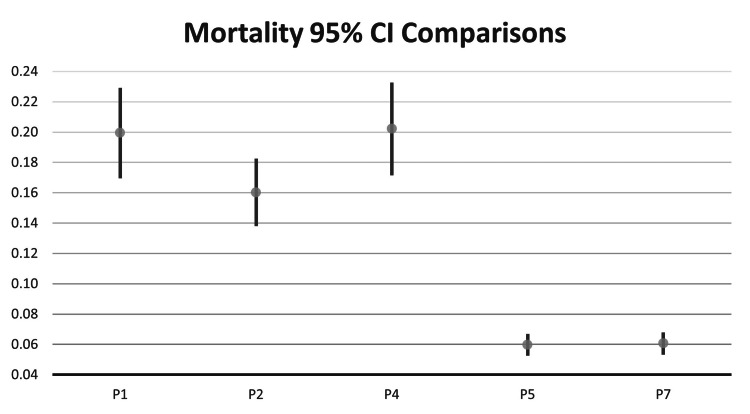
Mortality 95% confidence interval (CI) comparisons P1: Patients with both COVID-19 and diabetes; P2: Patients with COVID-19 and without diabetes; P3: Patients with COVID-19 and type I diabetes; P4: Patients with COVID-19 and type II diabetes; P5: Patients with diabetes and without COVID-19; P6: Patients with type I diabetes and without COVID-19; P7: Patients with type II diabetes and without COVID-19 This Figure is a visual representation of the data presented in Table 4. The 95% CIs of the sample proportion are represented by the top of the line (upper 95%) and bottom of the line (lower 95%). The sample proportion (mortality N over the total number in each subset) is represented in decimal format on the Y-axis. The X-axis lists each subset analyzed.

Statistically significant differences in mortality between subsets were calculated using a two-sample proportion test (Table 5). The mortality rate for P1 was 19.94% (137/687) with a standard deviation (SD) of 1.52%, and a 95% CI between 16.95% to 22.93%. For P2, the mortality rate was 16.03% (167/1042) with an SD of 1.14%, and a 95% CI between 13.80% to 18.25% (Figure 1). 

**Table 5:** Analysis results of two-sample proportion tests P1: Patients with both COVID-19 and diabetes; P2: Patients with COVID-19 and without diabetes; P3: Patients with COVID-19 and type I diabetes; P4: Patients with COVID-19 and type II diabetes; P5: Patients with diabetes and without COVID-19; P6: Patients with type I diabetes and without COVID-19; P7: Patients with type II diabetes and without COVID-19 Mortality (N) represents the number of persons in each subset who expired. It is represented here out of the number of persons in each subset (the sample proportion), e.g., in P1, 137 out of 687 persons total expired. The 95% confidence intervals (CIs) represent the differences between the sample proportions (the proportions of the subsets that expired). Px represents mortality sample 1, and Py represents mortality sample 2.

Comparisons	Mortality sample 1 (N)	Mortality sample 2 (N)	Px-Py	95% CI for Px-Py	p-value
P1 vs. P2	137 of 687 19.94%	167 of 1042 16.03%	3.91%	0.19%-7.64%	0.0364
P1 vs. P5	137 of 687 19.94%	249 of 4164 5.98%	13.96%	10.89%-17.04%	<0.0001
P1 vs. P7	137 of 687 19.94%	242 of 3992 6.06%	13.88%	10.80%-16.96%	<0.0001
P2 vs. P5	167 of 1042 16.03%	249 of 4164 5.98%	10.05%	7.71%-12.39%	<0.0001
P2 vs. P7	167 of 1042 16.03%	242 of 3992 6.06%	9.96%	7.62%-12.31%	<0.0001
P4 vs. P2	134 of 663 20.21%	167 of 1042 16.03%	4.18%	0.40%-7.97%	0.0272
P4 vs. P5	134 of 663 20.21%	249 of 4164 5.98%	14.23%	11.09%-17.37%	<0.0001
P4 vs. P7	134 of 663 20.21%	242 of 3992 6.06%	14.15%	11.00%-17.29%	<0.0001

For P5, the mortality rate was 5.98% (249/4164), with a 95% CI between 5.26% to 6.70%. Comparing this with P1 mortality of 19.94% (95% CI: 16.95%-22.93%, 137/687), the p-value is < 0.0001 (Table 5). The difference in mortality rate between the two subsets is 13.96% (95% CI 10.89%-17.04%). For P2, the mortality rate was 16.03% (95% CI: 13.80%-18.25%), 167/1042). Comparing this to P1 mortality of 19.94% (95% CI: 16.95%, 22.93%, 137/687), the p-value is 0.0364, indicating that there is a statistically significant difference between the subsets.

When looking at the subsets that have type II diabetes specifically, P4 reported a 20.21% mortality rate (95% CI: 17.15%-23.27%, 134/663); this is in contrast with P7, wherein the mortality rate was 6.06% (95% CI: 5.32%-6.80%, 242/3992).

## Discussion

Diabetes rates continue to surge across the globe [1]. If the increase in youth diabetes cases continues at the current rate, the number of youth affected by type II diabetes will increase by 673% by 2060 in the United States [18]. This is highly concerning for a myriad of public health reasons, not the least of which is that patients with diabetes have an increased risk of mortality when infected with COVID-19. The cost and accessibility of routine vaccinations, including COVID-19 boosters, continue to prove a concern for uninsured patients in particular. Federal funding for uninsured patients to receive COVID-19 shots is set to run out at the end of 2024 [19]. COVID-19 vaccine effectiveness has been demonstrated to wane over time [20], and the current uptake of the bivalent booster shot remains under 20%. Reasons for vaccine hesitancy include concerns about vaccine side effects/safety, in addition to doubt that updated boosters will offer additional protection [21]. However, newer treatment options for COVID-19 and diabetes may reduce mortality and morbidity in the future. New GLP-1 agonist drugs are proving a valuable tool for treating type II diabetes. The SURPASS-2 (A Study of Tirzepatide (LY3298176) Versus Semaglutide Once Weekly as Add-on Therapy to Metformin in Participants With Type 2 Diabetes) trial showed that tirzepatide caused an absolute reduction in glycated hemoglobin (Hba1c) by an average of 2.3% in the 15 mg group [22]. The landmark EPIC-HR (Evaluation of Protease Inhibition for Covid-19 in High-Risk Patients) trial found that Paxlovid (nirmatrelvir-ritonavir) reduced the risk of progression of COVID-19 to severe infection by 89% vs. placebo [23]. 

Key results

The results largely support our initial hypothesis that patients with both COVID-19 and diabetes will exhibit greater rates of mortality than those with COVID-19 without diabetes. The primary objective of this study was to analyze the relationship between COVID-19 and diabetes by adding information about trends in rural communities, in addition to discovering any differences that exist between type I and type II diabetes. This study was able to corroborate previous research showing that diabetes and COVID-19 comorbidity increases mortality. However, the study was underpowered concerning type I diabetes patients, as only 24 participants were included in P3. Therefore, P3 and P6 subset results were not included in further individual analysis, but the participants with type I diabetes were included along with type II diabetes participants within the P1 and P5 subsets.

A 2022 CDC study of COVID-19 patients in Pennsylvania, United States, found that patients with COVID-19 and type II diabetes were more likely to have elevated troponins (odds ratio (OR)= 1.87) and were at an increased likelihood of ICU admission (OR = 1.21). However, these associations were not significantly affected by the urbanicity of the patient area, and the study was not able to find a statistically significant association between type II diabetes and overall mortality. This same study also found that pre-pandemic ED visits were higher in urban areas vs. rural areas [24]. However, another study in North Carolina, United States, found that COVID-19 patients in rural areas were more likely to die or be discharged to hospice (16.5%) vs. patients in non-rural areas (13.3%). The 16.5%mortality/discharged to hospice subset is closest to the 16.03% mortality rate of the P2 subset [25]. A study of predominantly black COVID-19 patients in New York City found that diabetes significantly elevated the risk of mortality (adjusted relative risk (aRR) = 1.28).** **Overall mortality was 38.58% for those with diabetes and 27.25% for those without. This was much greater than the mortality rates shown in our study of 19.94% for P1 and 16.03% for P2. Age and coronary artery disease were found to increase COVID-19 mortality more so in those with diabetes than those without [26]. 

COVID-19 increases the risk of developing diabetes; one study found those infected were 2.35 times more likely to be diagnosed with diabetes in the 90 days following infection [27]. Other studies have investigated the relationship between hyperglycemia and COVID-19 on the molecular level. COVID-19 infection drives hyperglycemia by infecting hepatocytes and causing gluconeogenesis. COVID-19 viral entry is mediated in part through ACE2 and GRP78 (glucose-regulated protein 78) proteins [28]. ACE2 receptor levels are increased in diabetic patients [10]. Additionally, hyperinflammation via increased soluble urokinase plasminogen activator receptor (suPAR) levels, and hyperglycemia were found to drive increased COVID-19 mortality. This same study also found the risk of mortality was 1.23 times greater in patients with diabetes and COVID-19 infection than in those without diabetes [29]. Not only is the number of patients with diabetes increasing globally, but more patients are presenting with diabetic ketoacidosis at the time of diagnosis. Recent studies indicate that the COVID-19 pandemic may have accelerated this trend. A multi-center study across Europe and the United States found that in 2020, the rate of diabetic ketoacidosis (DKA) at the time of type I diabetes diagnosis in children was 39.4% vs. an average of 27.3% from 2006 to 2019 [30]. Similar results have been replicated in settings across the globe. A 2023 study from the University Hospital of Farhat Hached in Tunisia found a 48.17% increase in new-onset DKA since the start of the COVID-19 pandemic, with a corresponding increase in the diagnosis of type I diabetes by 50% and type II diabetes by 44% [31]. 

Limitations

Our study included data from the beginning of the pandemic until the end of 2021, including periods before and after vaccines were made widely available. Both vaccinated and unvaccinated persons with diabetes were included in the study. The effect size of the relative increase in mortality seen with diabetes and COVID-19 comorbidity may be either more or less pronounced in a vaccinated population. Over the course of the study period, the dominant circulating COVID-19 strain changed significantly, causing changes in virulence and mortality. It is possible the effect size seen would be greater or lesser depending on the strain of COVID-19 infection. The vast majority of patients included in the study were Caucasian. This is in line with the area served by Freeman Health and the population characteristics of the rural Midwest in the United States. The vast majority (84.6%) of the population of Joplin, the location of the largest Freeman Health hospital, is Caucasian [11]. Furthermore, due to the retrospective nature of the study, results may not be representative of the population as a whole.

Future studies on the subject should include larger sample sizes of patients with type I diabetes and COVID-19 to determine if type I diabetes diagnosis has a statistically significant effect on COVID-19 mortality. Variables such as sex, race, age, health insurance type, vaccination status, and Hba1c level should be analyzed for their impact on patient outcomes. While many of these variables were beyond the scope of our initial study, we intend to investigate them with greater detail in future studies.

## Conclusions

This study was conducted in a single health system, which helps to mitigate differences in patterns of care seen between institutions. Furthermore, this study took place within a rural community hospital patient population which is not as frequently studied as larger urban academic center patient populations. Our sample consisted of 19,323 admissions, adequately powering it to see small statistical differences of a few percentage points between the majority of subsets. This study focused on hospitalized patients and did not include patients who tested positive but did not require hospitalization. The results of our study demonstrate that patients with type II diabetes are more likely to die of COVID-19 infection than patients without, adding to the growing body of work relating to risk stratification of patients with diabetes and COVID-19. We hope that our study can help inform the conversation about these issues between physicians and their patients, especially in rural areas.
